# Still-Birth Certificates

**Published:** 1908-03-14

**Authors:** 


### STILL-BIRTH CERTIFICATES.

Sir,—I notice in the papers an account of an inquest on a child which was "killed" in the interests of the mother, the verdict being, "Necessarily destroyed to save the mother's life." In such cases there is seldom any necessity for an inquest, and I would like to ask if, in the event of the child being born dead, as it usually is, it would be justifiable to give a certificate of still-birth without mentioning the causes of the "still-birth"? Still-birth Certificate forms, unlike Death Certificate forms, make no provision for such particulars.

Yours faithfully, H.

